# Supplementary material for: Toxicological Assessments of a Pandemic COVID-19 Vaccine—Demonstrating the Suitability of a Platform Approach for mRNA Vaccines
Source: Vaccines (Basel). 2023 Feb 11;11(2):417. doi: 10.3390/vaccines11020417 (PMC9965811; doi:10.3390/vaccines11020417)

Supplemental Tables

Table S1 Dose Volumes Administered

Control or Test Article	Dose (µg RNA)	Total Dose Volume (µL)	Number of Sites x Dose Volume per Site
PBS/Sucrose	0	200	2 x 100 µL
BNT162b1	30	60	1 x 60 µL
BNT162b1	100	200	2 x 100 µL
BNT162b2 (V8)	100	200	2 x 100 µL
Saline	0	60	1 x 60 µL
BNT162b2 (V9)	30	60	1 x 60 µL
BNT162b3	30	60	1 x 60 µL

Table S2. Injection Site Draize Scoring

Score	Scoring Criteria
Edema	
0	No edema
1	Very slight edema (barely perceptible)
2	Slight edema (edges of area well defined by definite raising)
3	Moderate edema (raised approximately 1 mm)
4	Severe edema<br>(raised more than 1 mm and extending beyond area of exposure)
Erythema	
0	No erythema
1	Very slight erythema (barely perceptible)
2	Well-defined erythema
3	Moderate to severe erythema
4	Severe erythema (beef redness)
Induration or hardening[a]	
0	No induration/hardening
1	Very slight induration/hardening (barely perceptible)
2	Slight induration/hardening
3	Moderate induration/hardening
4	Severe induration/hardening
a. Induration or hardening was only recorded in Study 1.	

Table S3. Hematology parameters

Hematology Evaluation	
Red Blood Cells (RBC)<br>Red Cell Distribution Width (RDW)<br>Hemoglobin (HGB)<br>Hematocrit (HCT)<br>Mean Cell Volume (MCV)<br>Reticulocytes (RETIC)	Mean Platelet Component (MPC); Study 1 only<br>Mean Platelet Volume (MPV)<br>Mean Cell Hemoglobin (MCH)<br>White Blood Cells (WBC)<br>Mean Cell Hemoglobin Concentration (MCHC)<br>White Cell Differential<br>Platelets (PLT)<br>Plateletcrit (PCT); Study 1 only<br>Platelet Distribution Width (PDW); Study 1 only

Table S4. Coagulation parameters

Coagulation parameters	
Activated Partial Thromboplastin Time (APTT)<br>Prothrombin Time (PT)	Fibrinogen (FIB)

Table S5. Clinical Chemistry Parameters

Clinical Chemistry Evaluation	
Alanine Aminotransferase (ALT)<br>Albumin (ALB)<br>Aspartate Aminotransferase (AST)<br>Albumin/Globulin Ratio (AG)<br>Alkaline Phosphatase (ALP)<br>Bilirubin, Total (TBIL)<br>Blood Urea Nitrogen (BUN)<br>Calcium (CA)<br>Chloride (CL)	Cholesterol (CHOL)<br>Creatinine (CREA)<br>Creatine Kinase (CK); Study 1 only<br>Gamma Glutamyltransferase (GGT)<br>Globulin (GLOB)<br>Glucose (GLUC)<br>Lactate Dehydrogenase (LDH); Study 1 only<br>Phosphorus (PHOS)<br>Potassium (K)<br>Sodium (NA)<br>Triglyceride (TRIG)<br>Total Protein (TP)

Table S6. Urinalysis Parameters

Urinalysis	
Bilirubin<br>Blood/Hemoglobin<br>Clarity<br>Color; Study 2 only<br>Glucose<br>Urobilinogen (Study 1 only)	Nitrites; Study 1 only<br>Ketones<br>pH<br>Protein<br>Specific Gravity<br>Volume<br>Microscopic evaluation

Table S7. Tissue collection and organ weights

Tissue List	
Aorta	Lymph Node, Draining (Iliac)
Bone Marrow, Sternum	**Lymph Node, Inguinal**
Bone, Sternum or Femur	**Lymph Node, Mesenteric**
**Brain**	Macroscopic Findings
Cervix	Muscle, Skeletal
**Epididymis**	Nerve, Optic
Esophagus	Nerve, Peripheral
Eye	**Ovary**
**Gland, Adrenal**	Oviduct
Gland, Harderian	Pancreas
Gland, Lacrimal (Extraorbital)	Seminal vesicle
Gland, Mammary	Site, Injection
Gland, Parathyroid	Skin
**Gland, Pituitary**	Small Intestine, Duodenum
**Gland, Prostate**	Small Intestine, Ileum
Gland, Salivary	Small Intestine, Jejunum
Gland, Seminal Vesicle	Spinal Cord
**Gland, Thyroid**	**Spleen**
Gut-Associated Lymphoid Tissue	Stomach
**Heart**	**Testis**
Joint (knee)	**Thymus**
**Kidney**	Tongue
Large Intestine, Cecum	Trachea
Large Intestine, Colon	Ureter
Large Intestine, Rectum (Study 1 only)	Urinary Bladder
Larynx	Uterus
**Liver**	Vagina
**Lung**	
Lymph Node, Cervical (Study 1 only)	

Bolded organs represent the organs for which organ weights were collected in Study 1 and/or Study 2. Larynx was collected, but not examined.

Table S8: Test article-related clinical pathology findings (Ratio Relative to Control Mean)

Study		Study 1	Study 1	Study 1	Study 1	Study 1	Study 1	Study 2	Study 2	Study 2	Study 2
Sex		Male	Male	Male	Female	Female	Female	Male	Male	Female	Female
Test article		BNT162b1	BNT162b1	BNT162b2 (V8)	BNT162b1	BNT162b1	BNT162b2 (V8)	BNT162b2 (V9)	BNT162b3	BNT162b2 (V9)	BNT162b3
Parameter	Dose (µg/dose day)	30	100	100	30	100	100	30	30	30	30
ALB	Day 4	0.96x	0.93x	0.91x	0.92x	0.89x	0.87x	0.93x	0.92x	0.86x	0.90x
ALB	Day 17	0.96x	0.96x	0.94x	0.90x	0.89x	0.89x	-	-	0.85x	0.86x
GLOB	Day 4	1.16x	1.09x	1.07x	1.10x	1.08x	1.04x	-	-		1.05x
GLOB	Day 17	1.19x	1.26x	1.23x	1.18x	1.14x	1.18x	1.10x	1.07x	1.04x	-
GLOB	Day 38*	-	-	-	-	-	-	1.08x	-	1.06x	1.07x
AG	Day 4	0.83x	0.85x	0.85x	0.84x	0.83x	0.84x	0.90x	0.90x	0.86x	0.85x
AG	Day 17	0.81x	0.76x	0.76x	0.76x	0.78x	0.76x	0.89x	0.89x	0.82x	0.85x
AG	Day 38	-	-	-	-	-	-	-	-	0.91x	-
A2M	Day 4	36x	54x	54x	18x	91x	75x	20x	34x	3.3x	4.2x
A2M	Day 17	44x	279x	217x	45x	168x	121x	71x	128x	16x	18x
A1AGP	Day 4	5.9x	7.0x	6.9x	4.7x	5.6x	5.6x	9.4x	13x	8.0x	7.0x
A1AGP	Day 17	8.7x	19x	21x	8.9x	19x	16x	39x	42x	16x	17x
FIB	Day 17	2.6x	2.9x	3.1x	2.5x	2.6x	2.6x	2.4x	2.4x	2.5x	2.6x
HGB	Day 4	-	-	-	-	-	-	0.94x	0.93x	0.93x	0.93x
HGB	Day 17	-	0.89x	0.91x	0.90x	0.86x	0.87x	0.91x	0.93x	0.90x	0.89x
RDW	Day 17	-	-	-	-	-	-	1.21x	1.18x	1.18x	1.18x
RDW	Day 38	-	-	-	-	-	-	1.13x	1.12x	1.21x	1.23x
RETIC	Day 4	0.56x	0.37x	0.28x	-	-	0.52x	0.27x	0.27x	0.43x	0.44x
RETIC	Day 17	-	-	-	-	-	-	-	-	1.31x	1.20x
PLT	Day 17	-	0.75x	0.71x	-	0.66x	0.66x	-	-	-	-
WBC	Day 4	-	-	-	-	-	-	1.41x	1.28x	1.30x	1.43x
WBC	Day 17	-	1.82x	2.2x	1.79x	2.0x	2.1x	2.3x	2.2x	2.6x	3.0x
NEUT	Day 4	-	-	-	-	-	-	2.3x	2.0x	2.5x	3.1x
NEUT	Day 17	4.0x	5.5x	7.1x	5.9x	7.4x	7.8x	6.6x	6.5x	6.0x	7.0x
MONO	Day 4	-	-	-	-	-	-	1.83x	1.96x	1.89x	2.5x

MONO	Day 17	2.0x	1.78x	-	2.3x	2.1x	-	3.3x	3.6x	2.8x	3.1x
EO	Day 4	-	-	-	-	-	-	-	-	-	2.2x
EO	Day 17	2.1x	3.3x	5.2x	3.3x	5.4x	6.1x	2.5x	2.2x	3.2x	3.3x
BASO	Day 4	-	-	2.5x	-	-	-	1.88x	2.3x	1.89x	2.7x
BASO	Day 17	2.0x	2.1x	2.5x	2.2x	2.3x	2.1x	5.7x	6.3x	8.0x	10x
LUC	Day 4	-	-	4.0x	-	3.5x	4.2x	4.1x	4.0x	4.2x	4.4x
LUC	Day 17	2.7x	6.8x	7.9x	5.6x	8.1x	6.9x	8.0x	12x	13x	19x

- : no test article-related finding

* Day 38 was only included when a study group displayed a statistical difference as compared to control.

Supplementary Figure S1 Body weight in male (M) and female (F) Wistar Han rats immunized intramuscularly (IM) with a) BNT162b1 (30 µg or 100 µg), BNT162b2 V8 (100 µg) or buffer control; b) BNT162b2 V9 (30 µg), BNT162b3 (30 µg), or saline control.

a)

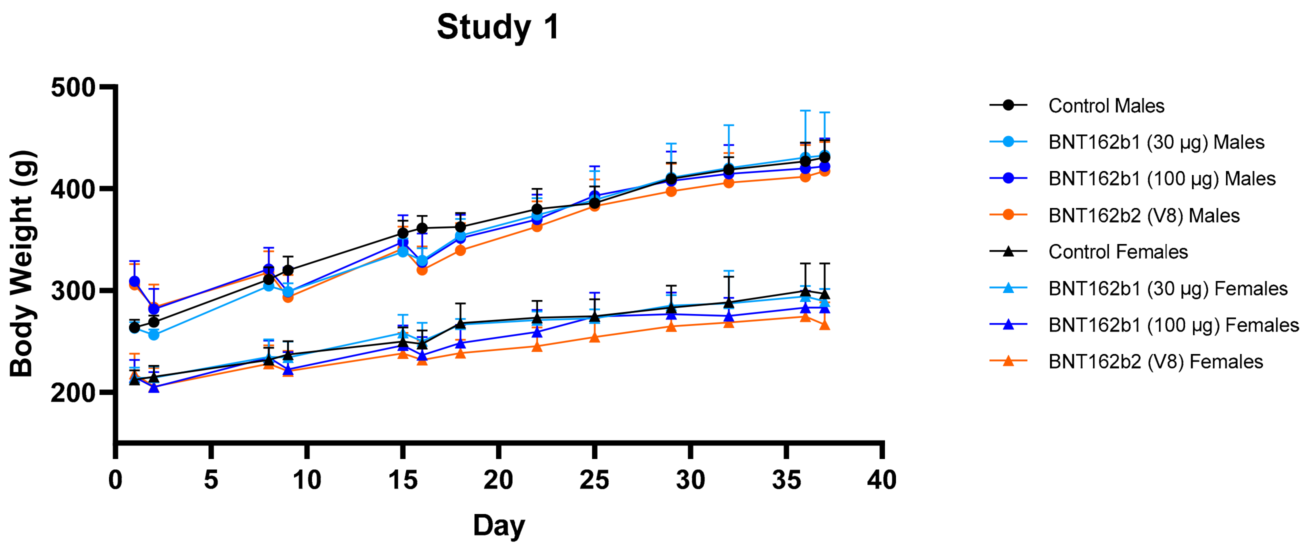

b)

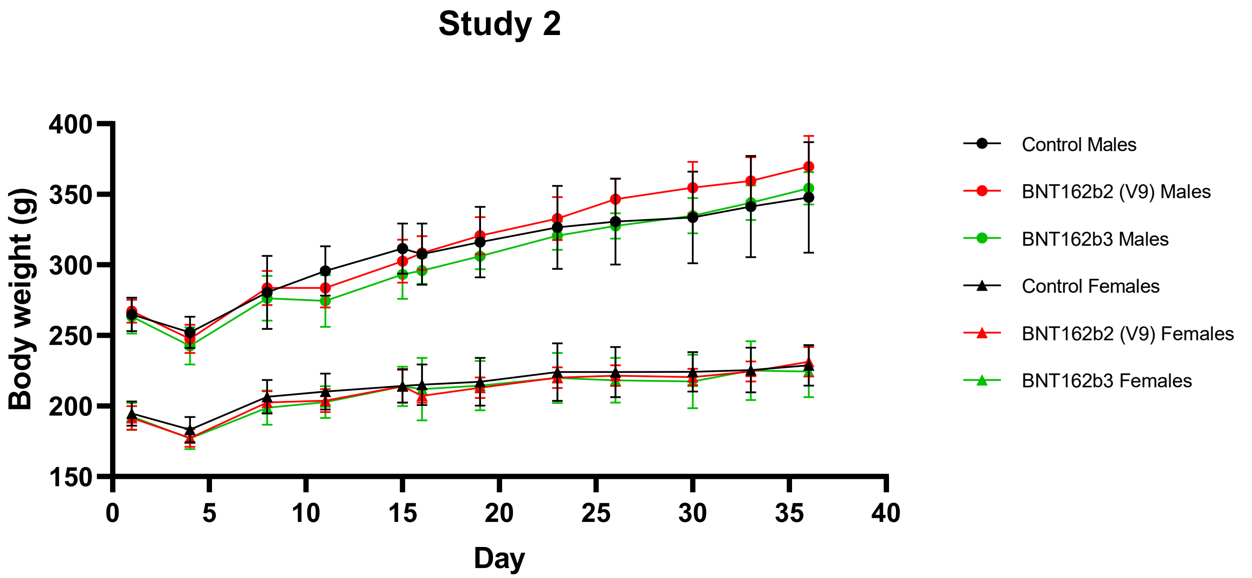

Supplement: Supplementary file 1 [file vaccines-11-00417-s001.zip › vaccines-2163984-supplementary.pdf]
